# Cluster headache – a study of 387 cases

**DOI:** 10.1186/1129-2377-14-S1-P51

**Published:** 2013-02-21

**Authors:** ME Nobre, PF Moreira

**Affiliations:** 1 Federal Fluminense University, Brazil

## Background

Cluster headache (CH) is a rare disorder with peculiar clinical characteristics. The most recent epidemiological study on suggested that CH is more common than previously thought and highlighted the long amount of time which elapses from onset of symptoms to diagnosis.

## Objectives

To conduct a nationwide study in order to describe the phenotype of CH in Brazil. We ultimately aimed to gather clinical data to support and refine the current diagnostic criteria.

## Methods

This study was conducted from January of 2009 to December of 2010. Patients with CH completed an online questionnaire (n = 658, and 387 of them were interviewed by phone. We obtained demographic information, initial assigned diagnosis, frequency, schedule, location, lateralization, history of smoking and alcohol consumption.

## Results

CH was more common in men (73.1%) than in women at a ratio of 2.7/1. Mean age at the time of assessment was 39.3 years. Most were white (82.7%). The first medical diagnosis received by these individuals was CH in only 28.7% of them. Most had episodic CH (66.9%) from the beginning, and 4.4% had episodic CH remitted from chronic. CH strictly on the right side occurred in 48.8% of the sufferers; strictly on the left occurred in 38.8%. Alternating unilateral CH for the same attack happened in 2.8% and for different attacks in 8.3%. Five individuals (1.3%) had bilateral CH. Tearing was the most frequent associated symptom (84.5%). Most attacks happened from midnight to 2am. A total of 55.8% of patients reported smoking, and 56.1% used alcohol more than twice per week.

## Conclusion

As expected, most sufferers were men, but with a male/female ratio smaller than previously reported. The proportion of chronic CH was higher than previously described. Some patients did not have strictly unilateral pain, and this possibility has recently been recognized, although not to the frequency found in our study. Delayed diagnosis was the norm, suggesting that continuous medical education on cluster headaches is necessary in order to relieve the incredible burden of these sufferers.
